# Comparison of Young’s Modulus of Continuous and Aligned Lignocellulosic Jute and Mallow Fibers Reinforced Polyester Composites Determined Both Experimentally and from Theoretical Prediction Models

**DOI:** 10.3390/polym14030401

**Published:** 2022-01-20

**Authors:** Maurício Maia Ribeiro, Miriane Alexandrino Pinheiro, Jean da Silva Rodrigues, Roberto Paulo Barbosa Ramos, Alessandro de Castro Corrêa, Sérgio Neves Monteiro, Alisson Clay Rios da Silva, Verônica Scarpini Candido

**Affiliations:** 1 Engineering of Natural Resources of the Amazon Program, Federal University of Pará—UFPA, Belem 66075-110, Brazil; mauricio_mribeiro@hotmail.com (M.M.R.); mirialexandrino@gmail.com (M.A.P.); 2 Materials Engineering Program, Federal Institute of Education, Science and Technology of Pará—IFPA, Belem 66093-020, Brazil; jean.rodrigues@ifpa.edu.br (J.d.S.R.); roberto.ramos@ifpa.edu.br (R.P.B.R.); alessandro.correa@ifpa.edu.br (A.d.C.C.); 3 Materials Science Program, Military Institute of Engineering—IME, Rio de Janeiro 22290-270, Brazil; snevesmonteiro@gmail.com; 4 Materials Science and Engineering Program, Federal University of Pará—UFPA, Ananindeua 67130-660, Brazil; alissonrios@ufpa.br

**Keywords:** natural fiber, tensile properties, micro-mechanics, vacuum-assisted hand lay-up

## Abstract

Mechanical properties of composites reinforced with lignocellulosic fibers have been researched in recent decades. Jute and mallow fibers are reinforcement alternatives, as they can contribute to increase the mechanical strength of composite materials. The present work aims to predict the Young’s modulus with application of continuous and aligned lignocellulosic fibers to be applied as reinforcement in polyester matrix. Fibers were manually separated and then arranged and aligned in the polyester matrix. Composites with addition 5, 15, and 25 vol% jute and mallow fibers were produced by vacuum-assisted hand lay-up/vaccum-bagging procedure. Samples were tested in tensile and the tensile strength, elasticity modulus, and deformation were determined. Results showed that the intrinsic Young’s modulus of the fibers was set at values around 17.95 and 11.72 GPa for jute and mallow fibers, respectively. Statistical analysis showed that composites reinforced with 15 and 25 vol% jute and mallow presented the highest values of tensile strength and Young’s modulus. The incorporation of 25 vol% of jute and mallow fibers increased the matrix Young’s modulus by 534% and 353%, respectively, effectively stiffening the composite material. Prediction models presented similar values for the Young’s modulus, showing that jute and mallow fibers might be used as potential reinforcement of polymeric matrices

## 1. Introduction

Natural lignocellulosic fibers polymer matrix composites have gained interest over the past few decades because of their availability, biodegradability, renewability, low density, low cost, high specific moduli, and environmentally friendly appeal [1,2,3]. Indeed, natural fiber-composite have been studied in past years to assess the possibility of their use in for non-structural and structural engineering applications [4,5,6] and knowing the chemical, physical, and mechanical properties of composites contribute to their application in several areas of engineering [3]. Factors such as exposure to radiation, temperature variation, chemical treatment, as well as fiber size and shape can influence the mechanical properties and dynamic mechanical behavior of composites reinforced with lignocellulosic fibers [7,8,9].

In study of [10] three types of composites with natural fiber reinforcements were researched: composites reinforced with hemp fabric in the 50% fraction and polyurethane resin matrix, composite reinforced with oak wood particles in polyester resin in six different dimensions of the particles in the volumetric fraction 25%, and hybrid composites reinforced with flax fibers in the volume fraction of 30%, with insertion of oak particulate, in polyester matrix. Among the different results obtained, the authors concluded that the tensile and rheological behavior depend on the size and disposition of fibers in the composite and on the type of matrix.

Croccolo et al. [11] used flax fibers in the production of composites in isophthalic (181EN2X) and vinyl ester (VEef220ST) matrices; the specimens were tested in tensile and it was observed that the composites reinforced with 50% of flax fibers in matrix 181EN2X overcame the pure matrix by 25.4% for tensile strength and 21.5% and for modulus of elasticity. Composites reinforced with 50% flax fibers in VEef220ST matrix surpassed the pure matrix by 11.2% and 37.7% for tensile strength and modulus of elasticity, respectively. The mechanical strength and rigidity of the composite with isophthalic resin overcame the composite with vinyl ester resin. Moreover, the isophthalic resin composites showed good adhesion between the matrix and the fiber.

Jute fiber and mallow fiber are relatively high-strength natural fibers with promising research and application potential. The work of Monteiro et al. [12] investigated the ballistic performance of plain-woven jute fabric-reinforced polyester matrix composites. The composites were produced with up to 30% by volume of fiber and orthophthalic polyester. Results showed that the composites exhibited ballistic behavior similar to that of aramid, also used as a component Multilayered Armor System.

Hanamanagouda et al. [13] studied mechanical properties of composites reinforced with 5, 10, 15, 20, and 25% by volume of jute fiber. The samples were produced by hot compression molding technique and then tested in impact, flexural, and tensile strengths. Results show that composites might be used in the manufacture of structural components.

The Izod impact and bend properties of composites reinforced with 10, 20, and 30 vol% of mallow fibers were determined [14]. The composites were molded by compression mixing aligned fiber and epoxy resin in a steel mold by. Results showed that composites fabricated with 20 and 30 vol% presented greater reinforcement capacity. Moreover, there was an interfacial detachment between the fiber and the matrix. The statistical analysis shows that composites reinforced with 30% by volume of mallow fiber presented the best performance.

It was reported that the polyester matrix composites reinforced with up to 40 vol% of mallow fabrics show exponential increase in tensile strength and linear increase in Young’s modulus [15].

The determination of tensile properties of manufactured composites are often carried out using some simple mathematical prediction models. These models give good approximations to predict tensile properties of continuous and uniaxially aligned fibers [1].

A model to calculate the Young’s modulus and tensile properties of 3D printed carbon-fiber-reinforced nylon matrix composite was presented by [16]. This model was elaborated from experimental data. The carbon fibers were aligned in the 0° direction in relation to the load applied in the tensile test. All samples tested exceeded the value of the Young’s modulus of the pure matrix of 0.92 GPa. In the proposed model, the mixing rule was converted so that the volumetric fraction was replaced by a percentage of the total area of carbon fibers.

There have been numbers of estimate models to predict mechanics properties of composites. The Rule-of-Mixture model (ROM) corresponds to the case when the applied load causes equal strains in the phases. The volume fraction of each phase is the principal element for pondering and determining the Young’s modulus of the composite. The ROM is considered an upper bound for the true elastic modulus [17]. The accuracy of this model is highly questionable because it cannot reflect the detailed constituent geometry and the dispersion structure among other features [18,19].

In the present work, the main prediction models are presented, starting with the ROM and followed by other theoretical prediction models. For comparison, experimental investigation was used to study the Young’s modulus of jute fiber- and mallow fiber-reinforced polyester composites incorporated with different fiber volume fraction. For the first time, a theoretical modeling of the Young’s modulus using six different types of mathematical models is presented. Furthermore, for the first time, an ample statistical analysis of the mechanical properties of polyester matrix composites reinforced with jute and mallow fibers is carried out.

## 2. Materials and Methods

### 2.1. Materials

The unsaturated terephthalate-based polyester resin (Arazyn AZ 1.0 #34) of average viscosity and the catalyst methyl-ethyl-ketone peroxide (MEK), PERMEC D-45, both supplied by Ara Química SA (São Paulo, Brazil), were used as the composite matrix. The polyester resin was mixed with 1.0 wt.% of catalytic hardener. The jute (*Corchorus capsularis*) and mallow (*Urena lobata*) natural lignocellulosic fiber used in this investigation were supplied by the “Companhia Têxtil de Castanhal do Pará” in the north region of Brazil. The fibers were manually separated and cut in lengths of 300 mm.

Table 1 shows the chemical composition of jute and mallow fibers.

### 2.2. Fiber Characterization

#### 2.2.1. Scanning Electron Microscope (SEM)

The surface of the fiber was observed using a scanning electron microscope (Tescan-Vega 3, Brno, Czech Republic) equipped with a 5-kV field emission electron source.

#### 2.2.2. Diameter and Density Determination

The fiber diameter was measured in a Carl Zeiss, model Stemi 508, camera AXIO-CAM 105 Color optical microscope (Carl Zeiss, Berlin, Germany) at 3 different positions along the axis of each fiber [4]. Fifty jute and mallow fibers average diameter were determined.

The density of the jute and mallow fibers were measured by the pycnometer method in an analytical balance with 0.0001 g of precision [25].

#### 2.2.3. Tensile Tests

The determination of the mechanical properties of the fibers was done by tensile test. The preparation of the specimen was performed according to ASTM C1557 [26]. Before the tensile test, the fibers were attached to 90 m/g^2^ paper using epoxy adhesive. The tensile tests were performed in an EMIC DL 10,000 (Instron, São José dos Pinhais, Paraná, Brazil) universal testing machine. Tensile tests were carried out on specimens with 40-mm gage length using the displacement control at a rate of 0.2 mm/min. For each type of fiber, 50 tests were performed, and the fibers were tested as received [27]. The cross-section of the fibers was considered circular, and their areas were used to calculate the tensile strength.

### 2.3. Composites Preparation

**Figure 1:**
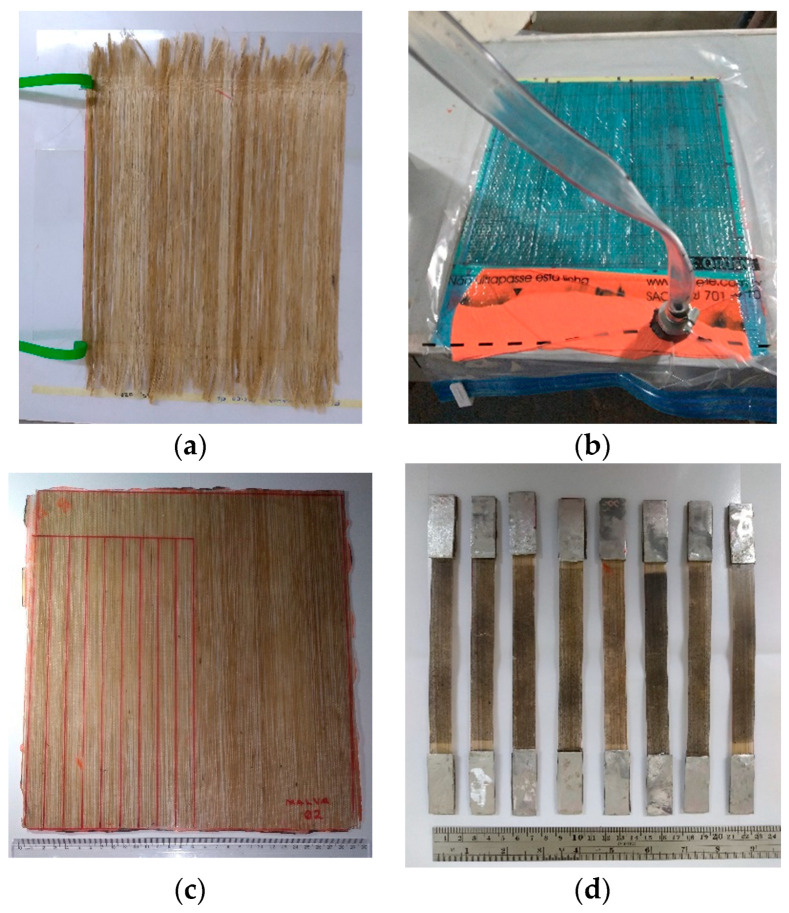
Manufacturing of fiber composites: (**a**) for mallow; (**b**) vacuum-bagging technique; (**c**) composite laminates produced; (**d**) specimens.

The jute and mallow fibers were accommodated continuous and aligned in a 300-mm-long mold (Figure 1a). Composites were produced by vacuum-assisted hand lay-up/vacuum-bagging procedure, stacking dry fiber ply layers onto a planar glass base to form a laminate. Resin was applied to the dry plies after hand lay-up, and then, the amount of resin needed for full impregnation was added, and vacuum was applied (Figure 1b). The fiber volume fraction precise content was evaluated on corresponding separate weighing amounts of the fiber layers. The resin used for production was carried out with the idea to use the least possible amount of resin needed for full impregnation. After the composite cured, the laminate of each type of composite was weighed, and an evaluation of the fiber volume fraction from the respective thickness of the layers compared to that of the whole composite was performed [20,21,22,23,24,25,26,27,28]. Values obtained were equal to 4.95%, 14.92%, and 24.48%, for jute fiber and 5.27%, 15.45%, and 23.90% for mallow fiber (Figure 1c). The dimensions of composites were 2 mm × 15 mm × 250 mm (Figure 1d).

### 2.4. Density Determination

Density and void measurements were carried out on composite with 50-mm diameter according to the Archimedean principle. The cured composite was then weighed in air and then again weighed in a liquid with a known density. The calculated density from measured values was reported in g/cm^3^ [29].

### 2.5. Tensile Test

**Figure 2:**
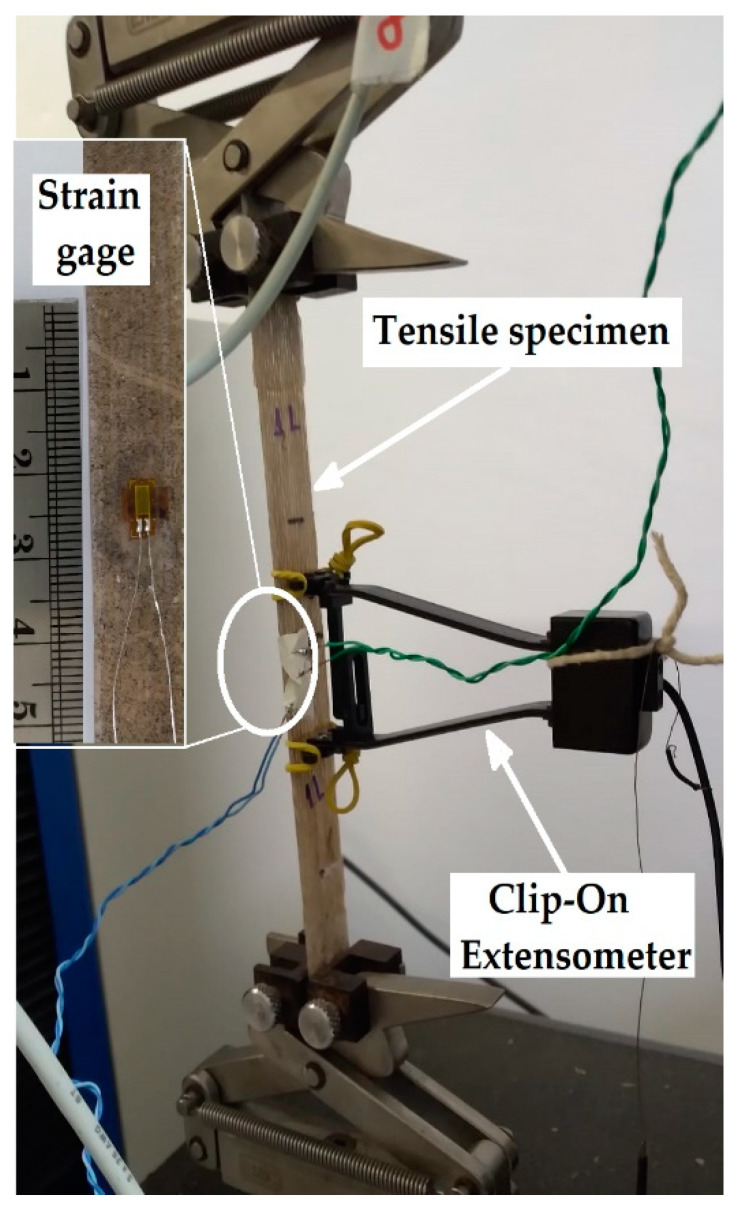
Experimental set-up with strain gage and extensometer for mechanical testing of composites (tensile test).

The tensile tests were carried out in eight specimens for each condition at 23 °C according to the ASTM 3039 [30] standard using an EMIC DL 10,000 (Instron, São José dos Pinhais, Paraná, Brazil) universal testing machine with a 5-kN load cell and Clip-On extensometer for displacement measurements during Young’s modulus determination (Figure 2). The crosshead speed was set as 2 mm/min for all tensile tests. To determine the mechanical properties in tensile, eight samples of each composition of the composites were tested.

### 2.6. Statistical Analysis

The statistical validation of the data was performed using the analysis of variance test (ANOVA), with a confidence interval of 95% (*p* < 0.05). The mean values were compared by the Tukey test.

### 2.7. Theoretical Prediction Models of Young’s Modulus

It is known that the mechanical properties of a composite depend on the intrinsic properties of its phases. In this way, when determining the individual properties of the reinforcement fibers and the matrix, we can apply to the existing micromechanical models that proved to be useful and reliable to calculate the intrinsic properties [31,32,33]. All models used in this paper are presented below and were used to predict the Young’s modulus of unidirectional continuous fiber-reinforced composites in the loading direction.

#### 2.7.1. Rule of Mixture Model (ROM)

The Rule of Mixtures model is the most commonly applied to represent Young’s modulus of unidirectional continuous fiber composite. It assumes that the interface between fiber and matrix is perfect and operates under iso-strain condition [1,34]. According to this model, Young’s modulus is calculated by the following equation:

(1) $$E_{c}=E_{f}V_{f}+E_{m}V_{m}$$

where *E* and *V* are Young’s modulus and volume fraction. The composite, matrix, and fibers are represented by the subscripts *c*, *m,* and *f*, respectively.

#### 2.7.2. Al-Quresh’s Model

Al-Quresh’s model is based on a combination of dispersed phases composed of fibers and particulates [35,36]. As such, by considering the principle of additivity, the equation of the rule of mixtures becomes:

(2) $$E_{c}=\beta E_{f}V_{f}+\lambda E_{m}V_{m}+\gamma E_{p}V_{p}$$

The factor *β* is associated with reinforcement efficiency in the case of parallel fibers and considered to have a value 1. The factor *λ* concerns the efficiency of the interfacial bond of the fiber by the matrix; in this paper, the value 1 was considered. The particulate is represented by the subscripts *p*, which is not considered in the present research.

#### 2.7.3. Madsen’s Model

In the Madsen’s model, the effect of porosity on the stiffness of plant fiber composites is demonstrated to be well simulated by including the factor (1 − *Vv*)^2^ in the ROM. The model assumes axial loading of the fibers as well as elastic stress transfer between matrix and fibers. The exponent 2 was considered in this paper. This value is denoted as the porosity efficiency exponent, where the author, in a broad range of plant fiber composite systems, found that *n* = 2 generally gives a good fit to the experimental data [37,38], using the following equation:

(3) $$E_{c}=(\eta_{0}.\eta_{l}.E_{f}V_{f}+E_{m}V_{m}){\left(1-V_{v}\right)}^{2}\;$$

The *η*_0_ is fiber orientation efficiency factor and is calculated for some typical fiber orientation distributions. In case of unidirectional and loaded along fibers, it is equal to 1.

The factor *η_l_* is fiber length efficiency factor for composites with fiber aspect ratios above 50; in the case of the composites presented in this paper, it was set equal to 1.

The void volume fraction needs to be calculated using the following equation:

(4) $$V_{v}=1-\frac{m_{f}/\rho_{f\;}+\left(m_{c}-m_{f}\right)/\rho_{m}}{m_{c}/\rho_{c}}\;\;\;$$

where *ρ_f_*, *ρ_m_*, and *ρ_c_* are density of fiber, matrix, and composite, respectively. They were calculated from experimental data. Just like *m_f_*, *m_m_*, and *m_c_* are mass of fiber, matrix, and composite, respectively, they were determined experimentally. The porosity is represented by the subscript *v*.

#### 2.7.4. Shah’s Model

The modified ROM, which takes into account the effect of fiber obliquity in twisted yarn reinforcements, is given by Equation (5). This model proposed a twist angle at the yarn surface *α* where a single plant fiber is similar to a twisted yarn composite. Single plant fibers are a lignin-hemicellulose matrix reinforced by cellulose fibrils where the microfibrils are at an angle to the vertical axis [39]; the model is represented by:

(5) $$E_{c}=(cos^{2}\propto .\eta_{l}.\eta_{d}.E_{f}V_{f}+E_{m}V_{m}){\left(1-V_{v}\right)}^{2}$$

where *α* is twist angle at the yarn surface and, in this paper, has a value of 18°.

The *η_d_* is distribution efficiency factor and reinforcement orientation, and it is assumed that *η_d_* is unity. The factor *η_l_* is the fiber length efficiency factor for composites with fiber aspect ratios above 50, as is the case for the composites presented in this paper, which were set equal to 1.

#### 2.7.5. Halpin-Tsai Model

Halpin and Tsai developed a semi-empirical model for the prediction and determination of the Young’s modulus of continuous fiber-reinforced composites aligned [40,41]. This model has been widely used among the prediction models for Young’s moduli in composite materials [1,42]:

(6) $$E_{c}=E_{m}\;\left(\frac{1+\zeta \eta V_{f}}{1-\eta V_{f}}\right)$$

where the constants *η* and ζ are given by:

(7) $$\eta =\left(\frac{\frac{E_{f}}{E_{m}}\;-1}{\frac{E_{f}}{E_{m}}+\zeta }\right)$$

(8) $$\zeta =\left(\frac{2l}{d}\right)$$

such that ζ is a shape fitting parameter. The variables *l* and *d* are the length of the fiber in the direction of the load and diameter of fiber, respectively [1].

#### 2.7.6. Nielsen’s Model

Landel and Nielsen [43] modified the Halpin-Tsai model by assuming a factor to account for the fiber arrangement as well as fiber content, *ψ*, to enable prediction as given by the following equations:

(9) $$E_{c}=E_{m}\;\left(\frac{1+\zeta \eta V_{f}}{1-\eta \psi V_{f}}\right)$$

and

(10) $$\psi =1+\left(\frac{\;1-\emptyset_{max}}{\;\emptyset_{max}^{2}}\right).V_{f}$$

where *∅_max_* is the maximum fiber packing fraction. In this paper, the fiber arrangement for random packing of fibers was adopted: 0.82 [44].

## 3. Results and Discussion

### 3.1. Fiber Characterization

**Figure 3:**
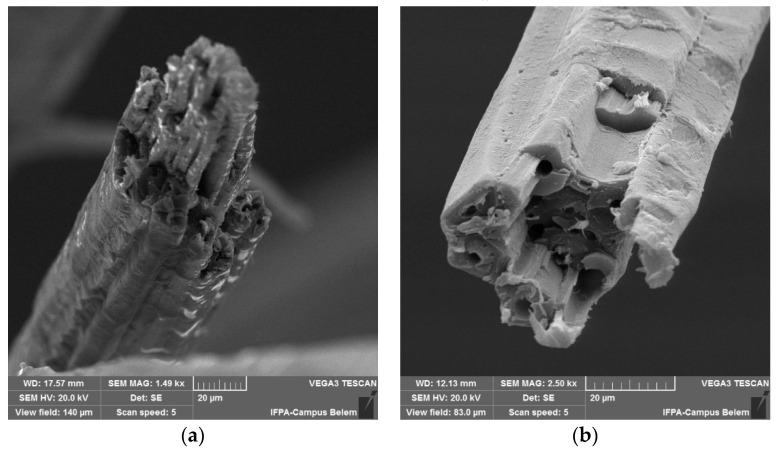
Scanning electron microscopy of fibers: (**a**) jute fiber; (**b**) mallow fiber. Magnification of 2.5.

SEM images of the jute and mallow fibers cross section are shown in Figure 3. It can be seen that it presents structures similar to those of other NFLs [4,5,6], with a lumen in the center and a structure that has an almost circular shape.

**Figure 4:**
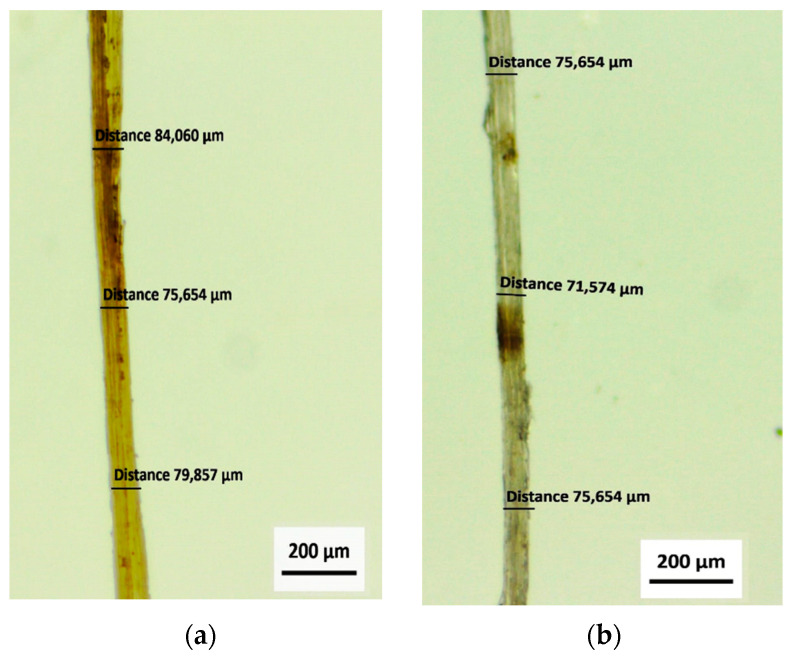
Optical microscopy showing specifics diameters: (**a**) for jute fiber and (**b**) mallow fiber.

Figure 4 shows optical microscopy of elementary fibers, where the values of the measurements of the diameters of the jute (Figure 4a) and mallow fibers (Figure 4b) are highlighted. Table 2 shows the average diameter, density, and mechanical properties of jute and mallow fibers.

Jute and mallow fibers had an average diameter of 78.00 ± 15.57 μm and 79.74 ± 18.15 μm and density of 1.482 ± 0.055 g/cm^3^ and 1.148 ± 0.068 g/cm^3^, respectively. The average ultimate tensile strength was 380.87 ± 89.32 MPa and 446.80 ± 104.47 MPa, and Young’s modulus was found to be 17.955 ± 6.57 GPa and 11.725 ± 4.09 GPa, respectively. Table 2 also shows that total strains were found to be 0.0292 ± 0.012 mm/mm and 0.0722 ± 0.030 mm/mm for the jute and mallow fibers, respectively.

One should notice that these experimental values are similar to those of jute fibers from the literature as shown in [45], where the presented density was 1.46 (g/cm^3^), tensile strength 393–800 (MPa), Young’s modulus 10–30 (GPa), and elongation 1.5–1.8 (%), and similarly for mallow fibers, [46]’s study presented a density of 1.37 (g/cm^3^), tensile strength of 160 (MPa), Young’s modulus of 17.4 (GPa), and elongation of 5.2 (%). The data provided in Table 1 were used in the prediction models to calculate the theoretical Young’s modulus.

### 3.2. Mechanical Properties of Composites

Table 3 presents the properties of jute and mallow fiber-reinforced composites. In this table, it might be seen that composite densities increase with the incorporation of jute and mallow fibers. It is also observed that the densities of the composites do not exceed the density value of the neat polyester matrix. The result of void volume fraction in Table 3 revealed that the manufacturing process of composites materials vacuum-assisted hand lay-up is a very effective method to prepare the composites.

**Figure 5:**
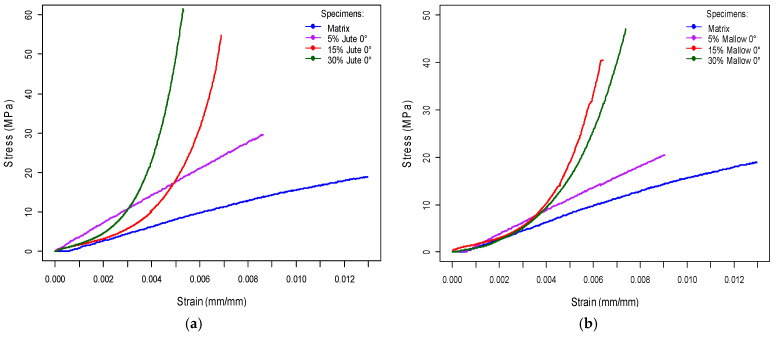
Typical stress versus strain curves: (**a**) for jute composites and (**b**) for mallow composites with different volume fraction.

Figure 5 shows the tensile curves of polyester matrix composites reinforced with 5, 15, and 25% volume of jute fiber and mallow fiber. Table 4 shows mechanical properties of both matrix and composites.

Results of Table 4 show that jute fiber-reinforced composites exhibit higher tensile strength, exceeding by 2%, 150%, and 166% the tensile strength of the neat polyester for addition of jute 5, 15, and 25 vol%, respectively. Similarly, the mallow fiber-reinforced composites also exhibit higher tensile strength, exceeding by 67% and 96% the tensile strength of the neat polyester for addition of mallow 15 and 25 vol%, respectively. Only for 5 vol% of mallow fibers was the matrix was superior by 23%. For the total strain in all cases, the neat polyester matrix shows higher results, where for 5, 15, and 25 vol% of jute fibers, the matrix was superior by 81, 80, and 83%, respectively, and for 5, 15, and 25 vol% of mallow fibers, the matrix was superior by 86, 85, and 85%, respectively. Similar results were obtained by [47].

Consequently, under this condition, the Young’s modulus for the 5, 15, and 25 vol% jute fiber-reinforced composites exhibit the highest values, exceeding by 86, 465, and 534% the stiffness of the neat polyester. Similarly, the Young’s modulus for the 5, 15, and 25 vol% mallow fiber-reinforced composites also exhibit the highest values, exceeding by 69, 248, and 353% the stiffness of the neat polyester, respectively.

### 3.3. Statistical Analysis

Statistical analysis of mechanical properties is presented in Table 5.

In Table 5, for maximum strength, one can see that the F calculated (12.89) is higher than the F critical value (3.411). For Young’s modulus, the F calculated (56.30) is higher than the F critical value (3.287). In the same way, for total strain, the F calculated (72.98) is higher than the F critical value (3.411). Thus, the hypothesis that the averages of the properties presented are equals, with a confidence level of 95%, is rejected. Because of the ANOVA results, the Tukey test was necessary in order to investigate if an increase in the amount of jute fiber volume fraction was more effective in causing significant changes in the mechanical properties in this composite.

Table 6 shows the results of Tukey test.

The minimum significant difference (m.s.d) is a value that can discriminate which treatment shows difference in its average values. Once the difference between the average values of groups, compared two by two, is higher than the m.s.d value, this pair is considered to be different. The m.s.d for maximum strength was calculated as 23.70, the m.s.d for Young’s modulus was calculated as 0.763, and for total strain, it was 0.0096.

Thus, it might be seen that the inclusion of continuous and aligned of jute fiber in polyester resin was essential to cause changes in mechanical properties. The J15% and J25% composites have indeed the highest tensile strength and Young’s modulus, which effectively stiffened the material.

For the results of maximum strength (MPa), Young’s modulus (GPa), and total strain (mm/mm) for composites reinforced with mallow fiber, the corresponding values for the ANOVA are shown in Table 7. The hypothesis that the averages of properties mechanical are equals, with a confidence level of 95%, is also rejected.

Results of ANOVA in Table 7 show that, for maximum strength, the F calculated (52.38) > F critical (3.411). For Young’s modulus, the F calculated (42.57) > F critic value (3.098). In the same way, for total strain, the F calculated (87.38) is higher than the F critical value (3.411). The Tukey test (see Table 8) was applied to discriminate which group showed significant differences in properties. The m.s.d for the maximum strength values was calculated as 7.11. The m.s.d for the Young’s modulus values was calculated as 0.553. For total strain, it was 0.0093.

M15% and M25% composites group, with 95% confidence level, exhibit the highest tensile strength and Young’s modulus. Thus, it might be seen that the inclusion of continuous and aligned mallow fiber in polyester resin caused changes in mechanical properties. On the other hand, no significant difference was found in the values of mechanical resistance between M5% composite and neat polyester matrix.

### 3.4. Theoretical Prediction Models of Young’s Modulus

**Figure 6:**
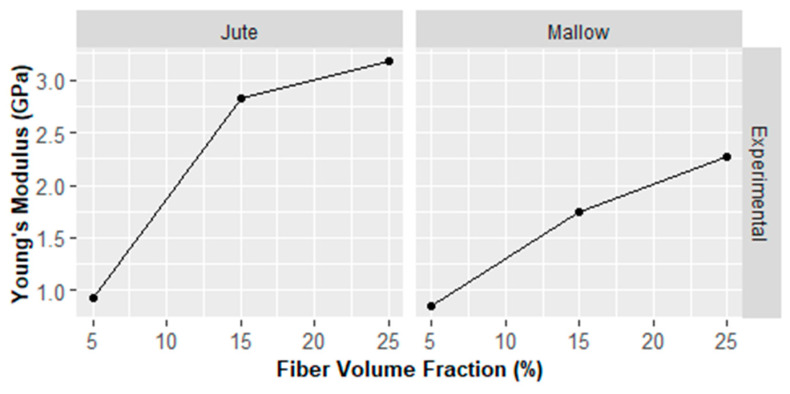
Young’s modulus of versus fiber volume fraction: experimental results.

Figure 6 shows the experimental variation of Young’s modulus of polyester matrix composites reinforced with jute fiber and mallow fiber as a function of fiber quantity.

It might be observed that the studied fibers participate in the increase of the mechanical properties of the composite materials. Indeed, the composites reinforced with jute fibers in volume fractions of 5, 15, and 25% showed increase of 0.934 ± 0.136 GPa, 2.831 ± 0.166 GPa, and 3.177 ± 0.794 GPa, respectively. The composites reinforced with mallow fibers also showed an increase in their mechanical property of 0.847 ± 0.080 GPa, 1.744 ± 0.179 GPa, and 2.271 ± 0.561 GPa, respectively. With an increase in the amount of fibers and a reduction in the amount of matrix, therefore, it is noted that due to is greater interfacial adhesion to fibers/matrix, there is a greater participation of fibers in the iso-deformation process. It should be noted that these experimental values are similar to previous works of Cavalcanti et al. [48]. Their research used 30 vol% jute fibers as reinforcement in an epoxy matrix and obtained an experimental Young’s modulus of 3.44 ± 0.20 GPa. Tanguy et al. [49] presented in their article 30% jute and flax fibers as reinforcement in a polypropylene matrix and obtained a Young’s modulus of 4.992 ± 0.242 and 4.227 ± 0.111 GPa, respectively.

Table 9 shows the variation of Young’s modulus predicted for composites reinforced with jute fiber and mallow fiber. The prediction models of the studied Young’s modulus showed increasing results, similar to the experimental ones.

**Figure 7:**
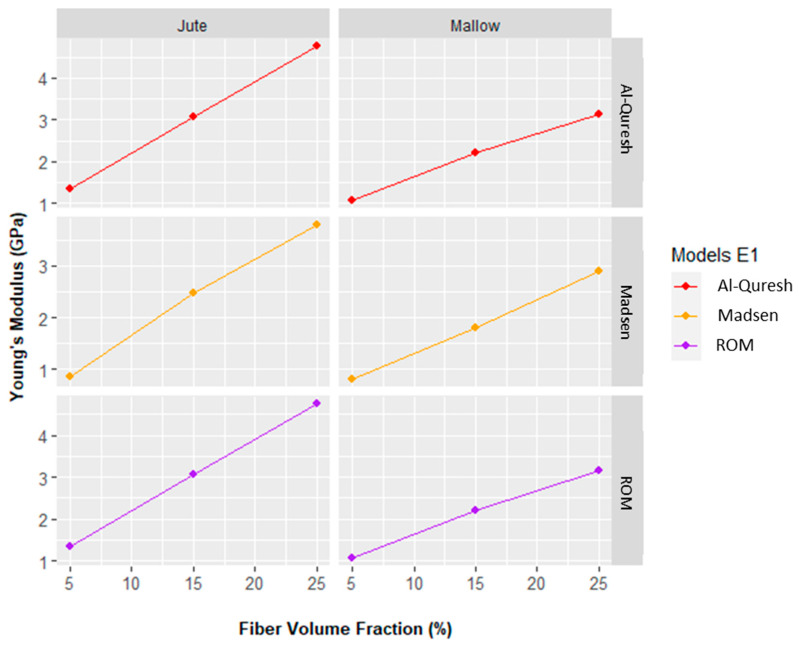
Young’s modulus of versus fiber volume fraction: Al-Quresh, Madsen, and ROM models.

Figure 7 shows the results of the mathematical modeling of the Young’s modulus of the studied composites.

Figure 7 shows that good fits between the theoretical and experimental values are obtained for the Madsen model. The difference between the Young’s modulus for jute fiber composites at 5, 15, and 25 vol% was 7, 13, and 19% and for the case of mallow composites were 4, 3, and 28%, respectively.

**Figure 8:**
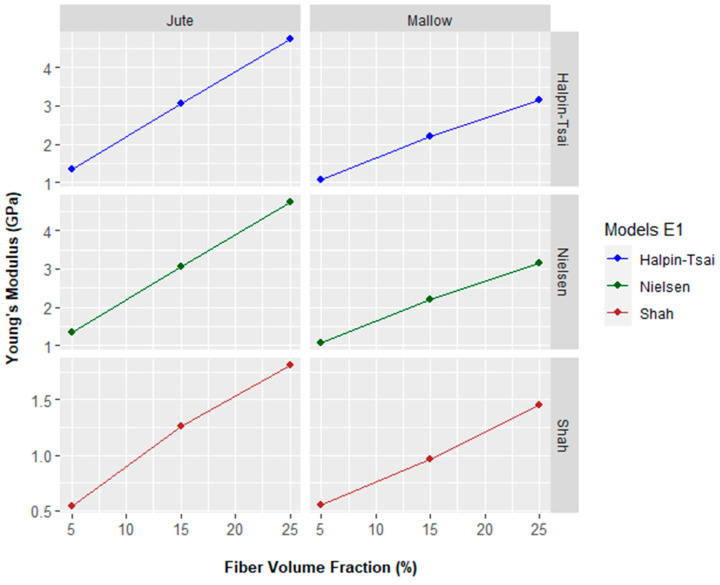
Young’s modulus of versus fiber volume fraction: Halpin-Tsai, Nielsen, and Shah models.

It can also be seen from Figure 8 that there is good agreement between in the ROM model and the Al-Quresh model, as both models showed the same Young’s modulus values. When comparing with experimental values, the disparity was 43, 9, and 50% for jute fibers and 26, 26, and 39% for mallow fibers.

Figure 8 shows the results of the mathematical modeling of the Young’s modulus of the studied composites.

Figure 8 shows that there is a difference between the Shah model and the experimental Young’s modulus. Thus, difference corresponds to a decrease of 42, 55, and 43% for composites reinforced with jute fiber and 36, 45, and 36% for composites reinforced with mallow fiber. Therefore, a discrepancy has also been observed between Shah model and experimental Young’s modulus.

Similar to the ROM and Al-Quresh models, it can also be seen from Figure 8 that there is good agreement between the Halpin-Tsai model and Nielsen model. Both models showed the same Young’s modulus values. When comparing with experimental values, the disparity was 43%, 8%, and 49% for composites reinforced with jute fiber and 25%, 25%, and 38% for composites reinforced with mallow fiber.

## 4. Summary and Conclusions

Polymer matrix and composite reinforced with continuous and aligned fibers of mallow and jute were prepared by the vacuum-assisted hand lay-up/vacuum-bagging method, varying the fiber volume fractions of 5, 15, and 25%. Based on the results obtained in this research work, the following conclusions can be drawn:

- This study reveals that the vacuum-assisted hand lay-up/vacuum-bagging method is very effective method to obtain composites with low void volume fraction;
- The prediction model that best fit the experimental results was the Madsen’s model; the difference between the Young’s modulus for jute fiber composites with 5, 15, and 25 vol% was 7, 13, and 19%, and for the mallow fiber with some compositions, it was 4, 3, and 28%, respectively;
- The ROM, Al-Quresh, Halpin-Tsai, and Nielsen prediction models showed similar results for Young’s modulus;
- Composites reinforced with 25 vol% of jute and mallow fibers presented higher tensile strength as well as Young’ modulus. This demonstrates that the inclusion of continuous and aligned of jute/mallow fiber in polyester resin can effectively stiffen the material; and
- It is concluded that the prediction models presented in this work display values close to experimental data. The work was successful, and we were able to confirm that jute and mallow fibers constitute a potential reinforcement of polymer matrices.

## Figures and Tables

**Table 1:** Chemical composition of natural fibers.

Fiber Type	Cellulose (%)	Hemicellulose (%)	Lignin (wt.%)	Wax (%)	References
Jute	50–72	12–20	8–13	0.5	[20,21,22,23]
Mallow	56–72	27–29	10–12	0.6	[24]

**Table 2:** Properties and result of tensile tests of samples fiber.

Fiber	Average Diameter (μm)	Density (g/cm^3^)	Tensile Strength (MPa)	Total Strain (mm/mm)	Young’s Modulus (GPa)
Jute Fiber	78.00 ± 15.57	1.482 ± 0.055	380.87 ± 89.32	0.0292 ± 0.012	17.955 ± 6.57
Mallow Fiber	79.74 ± 18.15	1.148 ± 0.068	446.80 ± 104.47	0.0722 ± 0.030	11.725 ± 4.09

**Table 3:** Properties of samples of fiber polyester composites.

Composite	Manufacturing Condition	Description	Fiber Volume Fraction (%)	Void Volume Fraction (%)	Density (g/cm^3^)
Neat Polyester Matrix	Hand lay-up method	PM *	0	NA **	1.249 ± 0.0310
Polyester/Jute fiber (0° unidirectional)	Vacuum-assisted hand lay-up/vacuum-bagging method	J5%	5	19.58	1.115 ± 0.0115
		J15%	15	10.34	1.154 ± 0.0136
		J25%	25	10.76	1.172 ± 0.0055
Polyester/Mallow fiber (0° unidirectional)		M5%	5	12.60	1.097 ± 0.0037
		M15%	15	9.70	1.112 ± 0.0136
		M25%	25	3.91	1.176 ± 0.0162

* PM polyester matrix; ** NA not applicable.

**Table 4:** Result of tensile tests of samples of fiber polyester composites.

Composite	Manufacturing Condition	Description	Fiber Volume Fraction (%)	Tensile Strength (MPa)	Total Strain (mm/mm)	Young’s Modulus (GPa)
Neat Polyester Matrix	Hand lay-up method	NA *	0	23.35 ± 4.46	0.0451 ± 0.0072	0.501 ± 0.084
Polyester/Jute fiber (0° unidirectional)	Vacuum-assisted hand lay-up/vacuum-bagging method	J5%	5	23.88 ± 4.22	0.0087 ± 0.0014	0.934 ± 0.136
		J15%	15	58.37 ± 2.53	0.0091 ± 0.0026	2.831 ± 0.166
		J25%	25	62.11 ± 2.12	0.0078 ± 0.0083	3.177 ± 0.794
Polyester/Mallow fiber (0° unidirectional)		M5%	5	17.93 ± 3.20	0.0064 ± 0.0024	0.847 ± 0.080
		M15%	15	39.11 ± 2.62	0.0066 ± 0.0023	1.744 ± 0.179
		M25%	25	45.82 ± 1.13	0.0069 ± 0.0009	2.271 ± 0.561

* NA not Applicable.

**Table 5:** Analysis of variance for composites reinforced with jute fiber.

Maximum Strength (MPa)						
**Source**	**Sum of Squares**	**Degrees of Freedom**	**Mean of Squares**	**F** **(calculated)**	**F critical**	***p*** **-value**
Between the groups	5044.28	3	1681.428	12.89	3.411	3.42 × 10^−4^
Inside the group	1696.32	13	130.486			
Total	6740.60	16				
Young’s Modulus (GPa)						
**Source**	**Sum of Squares**	**Degrees of Freedom**	**Mean of Squares**	**F** **(calculated)**	**F critical**	***p*** **-value**
Between the groups	23.69	3	7.898	56.30	3.287	2.14 × 10^−8^
Inside the group	2.10	15	0.140			
Total	25.80	18				
Total Strain (mm/mm)						
**Source**	**Sum of Squares**	**Degrees of Freedom**	**Mean of Squares**	**F** **(calculated)**	**F critical**	***p*** **-value**
Between the groups	0.0047	3	1.56 × 10^−3^	72.98	3411	8.70 × 10^−9^
Inside the group	0.0003	13	2.14 × 10^−5^			
Total	0.0050	16				

**Table 6:** Results obtained for differences between the average values for PM, J5%, J15%, and J25% after applying the Tukey test.

	Maximum Strength (m.s.d = 23.70)				Young’s Modulus (m.s.d = 0.763)				Total Strain (m.s.d = 0.0096)			
	MP	J5%	J15%	J25%	MP	J5%	J15%	J25%	MP	J5%	J15%	J25%
MP	0.00	0.53	**35.02**	**38.76**	0.000	0.433	**2.330**	**2.676**	0.0000	**0.0364**	**0.0360**	**0.0373**
J5%	0.53	0.00	**34.49**	**38.23**	0.433	0.000	**1.897**	**2.243**	**0.0364**	0.0000	0.0004	0.0009
J15%	**35.02**	**34.49**	0.00	3.74	**2.330**	**1.897**	0.000	0.346	**0.0360**	0.0004	0.0000	0.0013
J25%	**38.76**	**38.23**	3.74	0.00	**2.676**	**2.243**	0.346	0.000	**0.0373**	0.0009	0.0013	0.0000

**Table 7:** Analysis of variance for composites reinforced with mallow fiber.

Maximum Strength (MPa)						
**Source**	**Sum of Squares**	**Degrees of Freedom**	**Mean of Squares**	**F** **(calculated)**	**F critical**	***p*** **-value**
Between the groups	1844.06	3	614.687	52.38	3.411	1.61 × 10^−7^
Inside the group	152.55	13	11.734			
Total	1996.61	16				
Young’s Modulus (GPa)						
**Source**	**Sum of Squares**	**Degrees of Freedom**	**Mean of Squares**	**F** **(calculated)**	**F critical**	***p*** **-value**
Between the groups	10.00	3	3.333	42.57	3.098	7.18 × 10^−9^
Inside the group	1.57	20	0.078			
Total	11.57	23				
Total Strain (mm/mm)						
**Source**	**Sum of Squares**	**Degrees of Freedom**	**Mean of Squares**	**F** **(calculated)**	**F critical**	***p*** **-value**
Between the groups	0.0052	3	1.75 × 10^−3^	87.38	3.411	6.13 × 10^−9^
Inside the group	0.0003	13	2 × 10^−5^			
Total	0.0055	16				

**Table 8:** Results obtained for differences between the average values for PM, M5%, M15%, and M25% after applying the Tukey test.

	Maximum Strength (m.s.d = 7.11)				Young’s Modulus (m.s.d = 0.553)				Total Strain (m.s.d = 0.0093)			
	MP	M5%	M15%	M25%	MP	M5%	M15%	M25%	MP	M5%	M15%	M25%
MP	0.00	5.42	**15.76**	**22.47**	0.000	0.346	**1.243**	**1.770**	0.0000	**0.0387**	**0.0385**	**0.0382**
M5%	5.42	0.00	**21.18**	**27.89**	0.346	0.000	**0.897**	**1.424**	**0.0387**	0.0000	0.0002	0.0005
M15%	**15.76**	**21.18**	0.00	6.71	**1.243**	**0.897**	0.000	0.527	**0.0385**	0.0002	0.0000	0.0003
M25%	**22.47**	**27.89**	6.71	0.00	**1.770**	**1.424**	0.527	0.000	**0.0382**	0.0005	0.0003	0.0000

**Table 9:** Variation of predicted Young’s modulus for jute and mallow fiber polyester composites.

Composite	Fiber Volume Fraction (%)	Young’s Modulus (GPa)					
		ROM	Al-Quresh	Madsen	Halpin-Tsai	Nielsen	Shah
Polyester/Jute fiber (0° unidirectional)	5	1.337	1.337	0.865	1.332	1.334	0.540
	10	3.078	3.078	2.474	3.065	3.069	1.260
	15	4.751	4.751	3.784	4.733	4.738	1.809
Polyester/Mallow fiber (0° unidirectional)	5	1.064	1.064	0.812	1.061	1.062	0.546
	10	2.201	2.201	1.795	2.195	2.197	0.966
	15	3.147	3.147	2.905	3.138	3.141	1.453

## Data Availability

Not applicable.
